# The Portland Meeting

**Published:** 1905-04

**Authors:** 


### The Portland Meeting.

THE fifty-sixth annual meeting of the American Medical Association will be held at Portland, Oregon, July 11-14, 1905, under the presidency of Dr. Lewis S. McMurtry, of Louisville. On two occasions previously the association has met on the Pacific coast, both times at San Francisco. Other places of importance have grown up since then and it seems appropriate, if it be conceded again time to go beyond the Rocky Mountains, that Portland should be the place of meeting.

There are many ways or routes of travel that may be chosen from Buffalo to Portland. The Union Pacific from the Missouri river is always a favorite route, and in case one goes to San Francisco before the meeting it will be the natural line of travel. The American Surgical Association will hold its annual meeting in the city of the Golden Gate during the week anterior to the Portland gathering, and for those who attend this meeting the Union Pacific will afford the best means of travel outward bound. We understand the president, Dr. George Ben Johnston, of Richmond, will travel by the Northwestern and Union Pacific lines, returning by the Northern Pacific from Portland, visiting the Yellowstone park on the homeward trip.

It must not be forgotten, however, that there are other excellent lines. The Wisconsin Central railway speaks in our advertising columns in a businesslike fashion and offers an attractive way to reach the Northern Pacific. We understand that a special train is in contemplation for the New England contingent and other eastern travelers, including those from Buffalo who desire to avail themselves of this privilege.

Several other meetings will be held at Portland in conjunction with that of the American Medical Association; hence, everything considered, it is more than likely that the passenger traffic will be unusually large for a week or two before the official date given out. It is desirable, therefore, that those contemplating the trip should make early application to the railway offices.

It is of importance also that those expecting to visit Portland should make early reservation of rooms. Dr. Kenneth A. J. Mackenzie, chairman of the committee of arrangements, or Mr. H. C. Bowers, chairman of the hotel committee, at Portland, may be addressed on this subject.

---

THE hygiene of the mouth including the care of the teeth is of so much importance as a factor in preserving health, that physicians should ever and always exercise the greatest vigilance over their families in this respect. Besides, to keep the mouth clean and the teeth free of decay is one of the surest ways to prevent disease.

Not until recently has a proper dentifrice been manufactured that will serve all the purposes of mouth hygiene. Calox, a calcium dioxide, which gives up oxygen in the nascent state when brought into contact with the fluids of the mouth, is not only a germicide of absolute quality but also a powerful oxidising agent and antacid. In other words, it will *keep the mouth clean if* appropriately used,—a property which no other dentifrice that we have seen possesses,—chemically and hygienically clean we mean. Every physician should make himself familiar with calox. It is not promoted by an association of dentists interested in its sale, but is as legitimate a product of the chemical laboratory as any drug of the pharmacopeia.

---

Milo F. Jardin, of 104 Maple street, Buffalo, was arrested March 24, 1905, by detective sergeants Stoner and Holmes on a warrant charging him with violating section No. 153 of the health laws. The complainant is Dr. Henry R. Hopkins, chairman of the board of censors of the Medical Society of the County of Erie.

Jardin, it is alleged in the complaint, has been giving treatments for rheumatism and other ailments and also selling medicines.

---

Isaac A. Finch, of Buffalo, was convicted in the Erie County Court, in the latter days of February, of practising medicine without a license and was sentenced by Judge Emery, March 6, to serve three months in the penitentiary. Finch styled himself "doctor" and prescribed for patients, according to the complaint.

The Medical Society of the County of Erie, through its board of censors instituted the prosecution, it being learned Finch had put in a claim of about $100 against the estate of Mrs. Otilia Hoffman, who had died of cancer after being treated by Finch. The surrogate refused to allow the claim. Previously Mrs. Hoffman had paid $110 for treatment.

Subsequently Finch made application for a certificate of reasonable doubt, which was denied by Justice Lambert. Finch is now serving out his sentence.

---

Louisa Busch, of No. 277 Mills street, Buffalo, was arrested March 20, 1905, by detective sergeant Stoner, on a charge of practising medicine without a proper license. The complainant is Mrs. Mary Weil, of No. 428 Broadway. The accused was convicted before Judge Murphy, March 27, but sentence was suspended.

---

Dr. W. A. Bush, an osteopath of La Grange, Ind., was fined $25 for practising medicine without a license.—*Jour. Amer. Med. Assn.,* March 11, 1905.

